# Seasonal variation in mosquito abundance and environmental predictors in semi-pastoral southern Kenya: implications for endemic Rift Valley fever

**DOI:** 10.1186/s13071-025-07122-1

**Published:** 2025-11-27

**Authors:** Keli Nicole Gerken, Richard Rasto Olubowa, Tatenda Chiuya, Max Korir, Eric M. Fèvre, Andrew Stringer, Andy Morse, Matthew Baylis

**Affiliations:** 1 Institute of Infection, Veterinary, and Ecological Sciences, University of Liverpool, Liverpool, UK; 2 International Livestock Research Institute (ILRI), Nairobi, Kenya; 3 Centre for Development Research (ZEF), University of Bonn, Bonn, Germany

**Keywords:** Rift Valley fever virus, Mosquito ecology, Endemic transmission, Land-use, Irrigated cropland, Semi-pastoral

## Abstract

**Background:**

Ecological variables that vary across time and space shape mosquito populations, creating microenvironments that can become disease transmission hotspots. Rift Valley fever virus (RVFV), a priority zoonotic arbovirus, thrives in diverse conditions and has been detected in over 50 mosquito species. This diversity complicates efforts to identify the key vectors involved in transmission and highlights the need to understand how environmental conditions shape mosquito abundance in high-risk landscapes.

**Methods:**

This study investigated spatio-temporal variation in mosquito abundance across the semi-pastoral landscape of Loitokitok sub-county, Kajiado County, Kenya. Over a full year, inclusive of the 2023–2024 El Niño rains, repeated mosquito trapping events were conducted at households enrolled in a human clinical cohort study, with weather station data linked to each trapping event.

**Results:**

A total of 441 mosquitoes were captured across 39 trapping events, with an average of 11.3 mosquitoes per event. The highest rainfall occurred in November 2023, while mosquito abundance peaked in April 2024. Traps placed at households in cropland areas hosted significantly more mosquitoes overall and were associated with more *Anopheles* spp., predominantly *Anopheles gambiae* (Kruskal–Wallis *χ*^2^ = 6.9, *df* = 2, *P* = 0.03), while those in shrubland areas had more *Aedes aegypti* (Kruskal–Wallis *χ*^2^ = 11.9, *df* = 2, *P* = 0.002). Multivariable models showed that land use/land cover (LULC) consistently improved model fit, though temporal weather factors were stronger predictors. Weather conditions from the prior month better predicted mosquito abundance than weather conditions over shorter time frames, with temperature consistently included in top models and humidity outperforming rainfall as an additional covariate in the best-fit model that included LULC, temperature, and humidity.

**Conclusions:**

These findings highlight the role of weather patterns and LULC in shaping mosquito dynamics, with irrigated cropland likely creating persistent breeding sites and shrubland providing niches for *Ae. aegypti*. This emphasizes the need for targeted, community-driven vector control strategies to mitigate RVFV transmission risk and highlights the importance of altered agricultural landscapes in driving vector dynamics.

**Graphical abstract:**

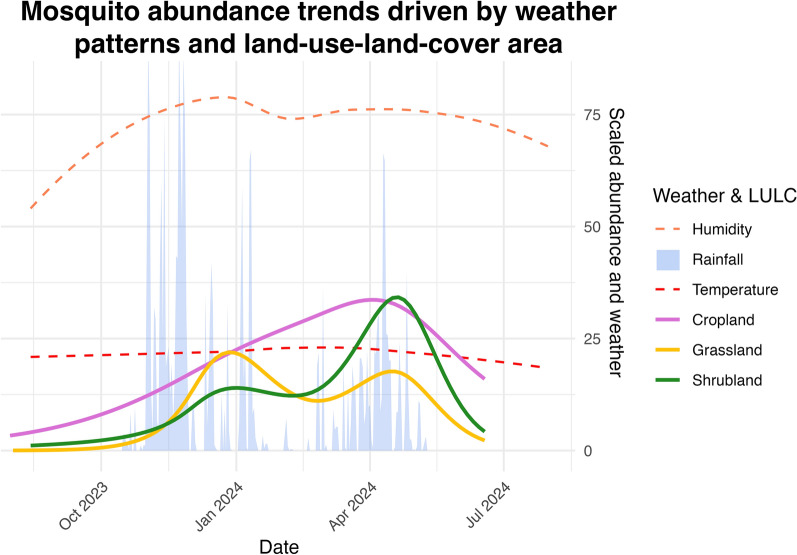

**Supplementary Information:**

The online version contains supplementary material available at 10.1186/s13071-025-07122-1.

## Background

Ecological variables impact mosquito populations, creating microenvironments that can lead to disease transmission hotspots [1]. Rift Valley fever virus (RVFV), a priority zoonotic arbovirus, thrives in diverse ecological conditions and mosquito species, complicating our understanding of the mosquitoes that drive transmission [2–4]. Investigating how ecological variables and land use influence mosquito populations contributes to a better understanding of the underlying drivers of endemic RVFV transmission.

RVFV primarily impacts domestic ruminants, leading to abortion and death of young animals in naïve areas. However, adult animals, especially in endemic areas, often do not have obvious clinical disease [5]. Humans become infected via mosquito bites or from direct exposure to the fluids and tissues of infected animals that may be asymptomatic [6, 7]. A study in northern Tanzania documented interepidemic transmission and spatial overlap of livestock and human exposure risk, with a human RVFV seroprevalence of 8.2% [8]. Livestock are inefficient at horizontally transmitting RVFV, necessitating arthropods for transmission and viral amplification [9]. Although other arthropods have demonstrated theoretical competence, mosquitoes are, by and large, the main vectors for RVFV [10, 11].

Unlike most arboviruses, RVFV has been detected in more than 50 different mosquito species during outbreaks and interepidemic periods [12]. Two hypotheses explain viral maintenance outside of large outbreaks: (1) emergence of dormant infected eggs from species capable of vertical transmission such as *Aedes* spp. and potentially *Culex* spp. [13] and (2) continuous low-level transmission in domestic and wild ruminants, amplified by ecological conditions [14, 15]. Traditionally, *Aedes* spp. were classified as primary vectors and *Culex* spp. as secondary vectors [16], but recent models show that RVFV can persist in some populations without reintroduction, challenging this dichotomy [17, 18]. While vertical transmission may increase the number of infected mosquitoes, ignoring maintenance via livestock undermines the role of *Culex* spp. in endemic transmission.

Data from field studies further highlight the complexity in extrapolating RVFV-positive mosquito data to an understanding of localized RVFV transmission. During a recent outbreak in Madagascar, RVFV was detected by reverse transcription polymerase chain reaction (RT-PCR) in 10 different mosquito species across nearby sites [19]. In Kenya’s 2007 outbreak, 10 mosquito species tested positive for RVFV, with species distribution varying by region: *Aedes* spp. dominated in the northeast, while *Aedes*, *Culex*, and *Mansonia* spp. were found in central (Baringo County) and coastal (Kilifi County) Kenya [16]. Even when a single mosquito species is the primary vector, arboviral diseases exploit ecological niches, thereby expanding range, seeding hotspots, and evading control measures, particularly under climate change scenarios [20]. The multi-level complexity of RVFV vectors makes this pathogen an ideal candidate for integrated vector management (IVM), a comprehensive approach targeting multiple mosquito life stages and risk categories.

Weather exerts a direct influence on mosquito abundance by affecting survival, development rates, and reproductive success, with extreme weather conditions acting as natural population constraints [21]. Mosquito populations increase through various mechanisms, including prolonged adult lifespans, accelerated larval and pupal development, and higher oviposition rates. Rainfall consistently correlates with an increased abundance of mosquitoes by creating breeding habitats, and this can translate to an increased risk of arboviral infections [22–24]. High humidity reduces potential evaporation of breeding sites and mitigates desiccation risk in adult mosquitoes, while temperature modulates developmental timelines and speeds up transitioning from larvae to adult [25, 26]. These correlates between weather and mosquito ecology are of course extremely well understood, but, despite this, the interactions between mosquito population dynamics and weather variability, including lag effects, remain poorly understood with respect to RVFV-endemic regions.

Human activity further modulates mosquito populations by altering natural habits, providing new breeding sites, and increasing available blood meal sources [27]. At the household level, anthropogenic change can select for mosquitoes that thrive around human spaces. For example, well-understood feeding, breeding, and resting patterns of mosquitoes are shifting in response to human presence, with *Aedes* spp. adapted to breed in small human-made containers of water and *Anopheles* spp. increasingly resting outdoors to avoid insecticide-treated nets indoors [28, 29]. At a broader scale, traditional pastoralism in semi-arid areas such as the Amboseli ecosystem in southern Kenya has shifted towards mixed crop farming, often involving irrigation and only periodic migrations [30, 31]. These land-use changes, along with drought-driven congregation of livestock and wildlife around limited water sources, may create persistent mosquito refuges and elevate exposure risk during a time when risk of vector-borne disease (VBD) is generally considered low [32, 33]. Semi-pastoral settings are indeed ecologically complex, shaped by land fragmentation, widespread livestock ownership, and changing mobility patterns [30, 31, 34]. These dynamics make such environments especially relevant for understanding how diseases like Rift Valley fever (RVF) persist under endemic conditions.

Given these complexities and the need to better understand vector dynamics in RVF-endemic areas, this study investigates the broader ecological factors influencing mosquito abundance. Rather than focusing on individual mosquito species, we assess broad environmental drivers of overall mosquito abundance, treating all species as potential contributors to RVFV transmission. Our findings support improved IVM strategies by predicting temporal risk and identifying ecological hotspots in endemic regions.

## Methods

This study aimed to identify spatio-temporal variation in mosquito abundance across the semi-pastoral landscape of Loitokitok sub-county in Kajiado County, Kenya. The study was nested within a human clinical cohort study at the household level. Over a full year, repeated trapping events for outdoor mosquitoes at households were carried out and parameters were recorded by weather stations. We assessed factors associated with increased mosquito abundance and temporal trends in weather.

### Study site overview

The Loitokitok sub-county is located at the base of Mount Kilimanjaro and borders Tanzania to the south, Chyulu Hills to the west, and the Amboseli ecosystem to the east. The climate is warm and semi-arid, with a mean temperature of 18.3 °C and average yearly precipitation of 433.0 mm, varying significantly by month (2.0–115.9 mm) (2022, CHIRPS: Climate Hazards Group InfraRed Precipitation with Station) [35]. The Loitokitok sub-county, located in Kajiado County, has experienced extensive land-use change since Kenya gained its independence in 1963, and the effects of climate change have resulted in varied rainy seasons and drought periods [36]. In the most recent census in 2019, the sub-county had a population of 117,737, of which 28,470 (24.2%) individuals were living in urban settlements. There were 47,058 registered households, and only 32,205 (70.7%) of these had proof of land tenure. Of all households in the sub-county, 29,501 (62.7%) were involved in farming, and there were slightly more households involved in livestock production (22,110, 47.0%) than in crop production (20,249, 43.0%). At the time of the census, of those households that were crop farming, 36.5% (7,400/20,249) used an irrigation system [37].

**Fig. 1:**
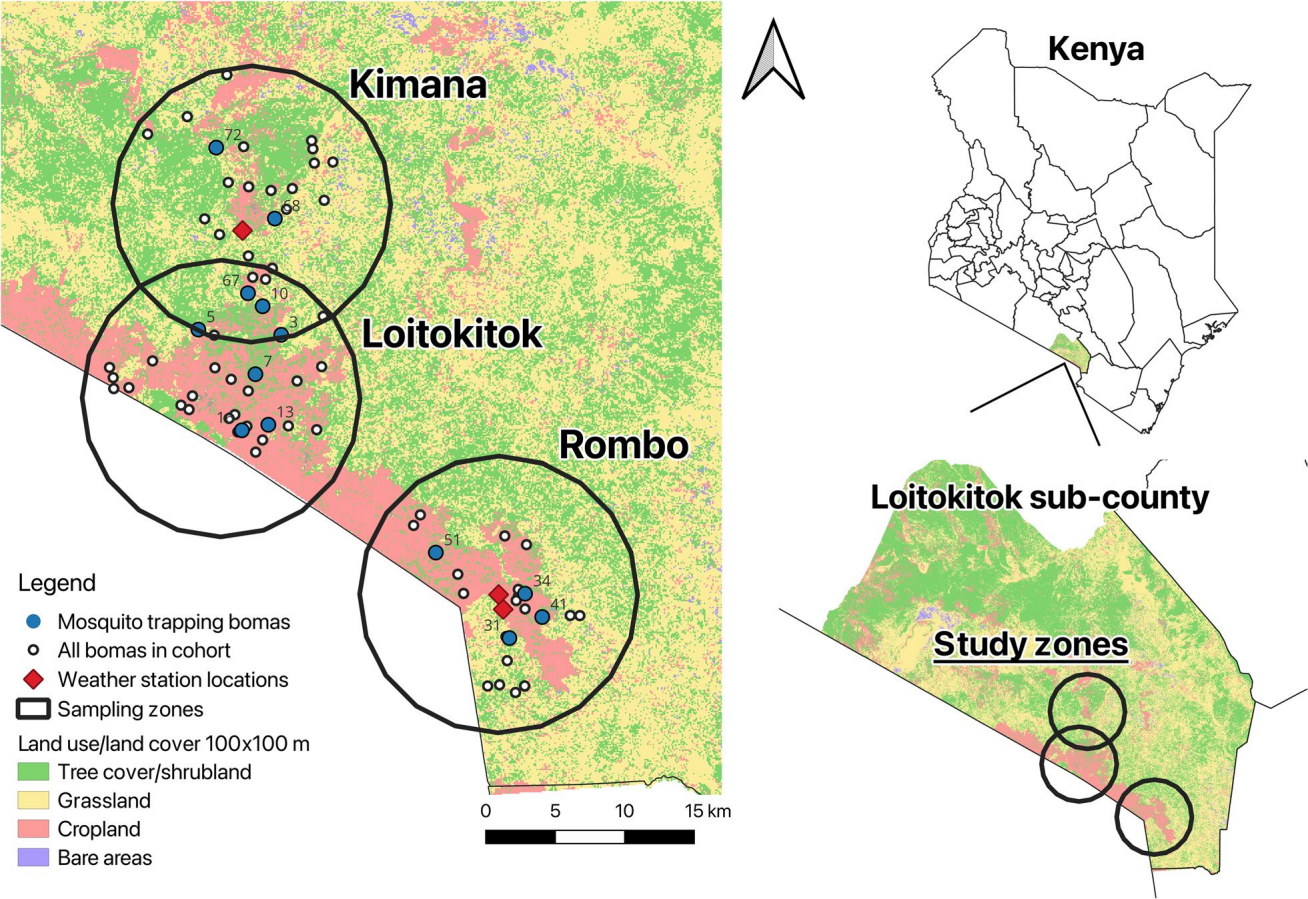
Overview of the Loitokitok sub-county study site in Kenya, including the sampling zones, all bomas enrolled in the cohort, mosquito trapping bomas, and weather station placements in the centres of Kimana and Rombo zones. m, metres

To assign sampling zones in Loitokitok sub-county, three zones, each with a radius of 10 km, were created around Kimana, Rombo, and Loitokitok market towns, all of which are within and serve the Loitokitok sub-county (Fig. 1). Households in this vector study were originally randomized for a human clinical cohort of 69 households, and vector trapping households were then purposefully selected based on road access and cooperation. Using the Google Open Buildings model (https://sites.research.google/gr/open-buildings/), buildings were identified and randomly selected in QGIS (version 3.32.3–Lima) after excluding town centres. Routes were mapped via Google Earth, and the field team, with local leaders, visited sites to assess eligibility. Households that consented were required to own at least five cattle, sheep, or goats.

### Classification of land use/land cover across the study site

To create a land-use/land-cover (LULC) map of the study site, a Sentinel-2 satellite image from 2022 was used to create land-cover classes for Loitokitok sub-county. The random forest approach was used within the Google Earth Engine platform. The three major LULC areas in the sub-county were grassland, cropland, and shrubland. Cropland patches are dispersed throughout the entire sub-county but are centred around towns (Figure 1). The LULC map was divided into a 100 × 100 m grid, and all participating households were assigned to a LULC area grid square. Although the classification was based on satellite imagery alone, we refer to this as LULC throughout this study, because the cover classes (e.g. cropland) also broadly reflect land use in this setting.

### Site selection, trapping event description, and data collection

At least three households from each study zone in each LULC were selected for mosquito trapping. Outdoor mosquitoes associated with shared livestock and human spaces were targeted. Trap placement was standardized as much as possible and was always within the household compound. Each trap was set off the ground about 1.5 m in a sheltered location, such as beneath a roofed structure or overhang. Traps were always placed at least 1 m away from livestock pens and away from high-traffic areas. We aimed to position each trap roughly equidistant from human sleeping quarters and animal enclosures; actual distances to both were measured and recorded for every trapping event. These placement rules were intended to maximize the likelihood of capturing mosquitoes shared by human–livestock spaces and maintain consistency across sites, which was balanced with protecting the trap and battery from rain, animals, and children. The trap was not moved for the duration of the trapping event, and when events were repeated in households, the trap was placed in the same location. Households were sampled between one and seven times.

Trapping events were scheduled to last between 48 and 72 h, and every 24 h the catch bag was retrieved, mosquitoes were sorted, and the battery on the trap was replaced with a fully charged battery. Day and nighttime mosquitoes were combined in this 24-h catch, and our analysis therefore reflects total abundance over 24 h. We deployed two different types of Biogents (BG) traps in this study: the BG-Counter 2, which connects to an industrial CO_2_ tank, and a standard BG trap, which was connected to a closed delivery system involving yeast and sugar fermentation that has been described by Ngenga et al. [38]. CO_2_ was the only bait used throughout the study with these two mosquito traps, and these two CO_2_ production mechanisms were assessed to catch an equivalent number of mosquitoes in a pilot study carried out in a semi-controlled environment away from the field study site on the campus of the International Livestock Research Institute (ILRI) in Nairobi, Kenya. Trapping events took place alongside other field activities, and hence, the traps were set and revisited at various times during daytime. The earliest time the trap was set was 8:30 and the latest was 18:28, and this discrepancy in total trapping time is accounted for in the data management and analysis described below.

The first field mosquito trapping event took place on 31st July 2023 and the last on 18th June 2024. Given that only one trap was placed on the last day of July, this event was included in the August aggregates for analysis. All mosquito sorting and counting were conducted by a trained research assistant and vector technician (RRO) who worked alongside the lead author (KNG) throughout the study, to ensure consistency in field implementation, mosquito handling, and morphological identification of mosquitoes.

When the catch bag was retrieved from the trap, it was transported to the laboratory within 2 h while mosquitoes were still alive. Mosquitoes were killed by placing the catch bag at 4 °C for 5 min. Once immobile, non-mosquito insects were removed, and mosquitoes were counted and sorted on a white tray by sex, genus, and feeding status. Where morphological identification allowed, the mosquito species was also recorded. The morphological identification followed guidelines from training led by the Kenya Medical Research Institute (KEMRI) and with keys published by Gilles and Coetzee [39]. Given the primary outcome of interest was mosquito abundance, we did not carry out any additional molecular identification of mosquitoes.

For each trapping event, a structured survey was completed that included data on potential larval breeding sites. These data included an assessment of standing water within the compound, open water containers, and irrigated cropland within 500 m. Potential breeding sources that were visible and accessible were visually inspected for mosquito larvae with a torch and small probe and systematically recorded in the dataset.

### Weather station data collection

To assess associations between mosquito abundance and weather, two weather stations were installed in secure central locations at health centres in Kimana and Rombo study zones (Fig. 1). Devices included the Ecowitt HP2551 (measuring temperature, humidity, wind, rainfall, and ultraviolet [UV] and solar radiation) and the Tinytag data logger (temperature and humidity), housed in a solar radiation shield (AcuRite 06054M). In Kimana, both were mounted on the same pole; in Rombo, they were ~300 m apart. Stations were elevated (~2.5 m) and positioned away from trees and buildings. Data were downloaded every  3 months and aggregated to daily and monthly values. This study focuses on rainfall (mm), temperature (°C), and relative humidity (% RH).

Weather data from August 2023 were unavailable as we did not have the weather stations at the start of the study, so values from August 2024 were used. This substitution was considered appropriate to maintain the same weather variable formatting and because August is consistently dry in this region, with minimal interannual variation in rainfall and temperature. This was confirmed by comparing remotely sensed CHIRPS mean daily rainfall data for the study site for August 2023 (5.3 mm) and August 2024 (5.1 mm), which was not significantly different (*t* = 0.09, *P* = 0.93). Further, average temperatures in August are also consistently recorded at 16.5 °C with little to no interannual variation. Indeed, temperature fluctuations are generally minimal in dry months [39].

### Data analysis

#### Univariable analysis and data visualization

For all univariable analyses, the outcome was the total number of mosquitoes per day, adjusted for total trapping time. This was calculated by multiplying the total catch by (24/total trapping time) and then log-transforming the result for statistical tests. Data were visualized using histograms, bar plots, and box plots. To test for statistical significance between the outcome and predictor variables, Wilcoxon rank-sum test was used for categorical variables with two groups and Kruskal–Wallis Chi-square tests when there were more than two groups; *t*-tests were used for continuous predictors. The number and proportion of mosquito species were examined and visualized over time using line and stacked bar plots.

#### Weather data aggregation

For each of the 39 mosquito trapping events, average temperature, average humidity, and total rainfall were calculated for the 2 weeks and 1 month prior to the event using data from the associated Ecowitt stations. As there was no weather station in the Loitokitok zone, data were extracted from the nearest station to the household.

Weather data were visualized by overlaying mosquito abundance plots. We hypothesized that month of trapping would serve as a proxy for weather and confirmed this by aggregating the average temperature and humidity (prior 2 weeks and 1 month) and rainfall (prior 2 weeks) for all categorical predictors associated with each data point.

#### Multivariable statistical modelling

The outcome variable for all multivariable modelling was the same as the univariable analyses, the total number of mosquitoes expressed as the total number of mosquitoes per day. Only biologically plausible predictors were considered in variable selection. Outcome data were visualized and inspected for normality and overdispersion (where variance > mean) to determine whether models using a Poisson or negative binomial distribution were more appropriate. As these models are designed for count data, the outcome variable was rounded to the nearest whole number.

Initially, only LULC and month were considered in multivariable models, separately, together, and as an interaction term with sampling month determined to represent a proxy for varied weather over time. The Akaike information criterion (AIC), deviance, and McFadden’s pseudo-*R*^2^, a commonly used likelihood-based analogue of the coefficient of determination (*R*^2^)  for generalised linear models, including  negative binomial models,  were tracked to compare model fit (Additional file 1) [40].

The month variable was replaced by the calculated weather data matched to each trapping event, and the MASS R package was used to fit negative binomial regression models with multiple predictors. Highly correlated weather variables were not considered together in the same model as determined by the Pearson correlation coefficient (*r*). Additional verification to confirm absence of multicollinearity was performed using variance inflation factors in R. R and RStudio (version 2024.12.0+467) were used to carry out all analyses and for creation of plots. QGIS (version 3.42.1-Münster) was used to produce the study site map.

## Results

### Trapping household-level mosquitoes across different land-use areas

Across 13 households and 39 trapping events, we caught a total of 441 mosquitoes. Descriptive statistics are presented in Table 1. The mean number of mosquitoes per trapping event was 11.3 (median = 10; range = 0–37). Trapping events lasted on average 51.3 h (median = 47, range = 40–94). The greatest influence on the total number of mosquitoes was the trapping month (Kruskal–Wallis *χ*^2^ = 24.5, *df* = 6, *P* = 0.0004), with April having the highest abundance.

**Table 1:** Descriptive statistics of ecological variables and statistical associations with the total number of mosquitoes adjusted for the total trapping time

Variable	Variable categories or measures of central tendency	No of trapping events in category or descriptive statistics	Total mosquitoes/24 h (mean, median)	*P*-value
Land use/land cover	Grassland	12	4.2, 3.5	0.14
	Cropland	15	7.7, 7.3	
	Shrubland	12	4.8, 4.2	
Study zone	Kimana	7	4.6, 3.4	0.49
	Loitokitok	24	5.7, 4.3	
	Rombo	8	6.8, 5.9	
Sampling month	August	5	0.9, 0.7	0.0004
	November	6	3.3, 3.3	
	December	7	7.8, 6.3	
	Feb	12	6.8, 5.2	
	April	3	13.9, 15.2	
	May	3	5.1. 3.9	
	June	3	1.8, 1.5	
BG trap type	Counter with CO_2_ tank	18	5.6, 4.5	0.82
	Standard BG with yeast	21	5.8, 4.6	
Household altitude (m)	Range	1093–1701		0.31
	Mean	1433		
	Median	1497		
	1st quartile; 3rd quartile	1297; 1573		
Distance BG trap to animal pen (m)	Range	1.1–16.3		0.23
	Mean	7.9		
	Median	5.8		
	1st quartile; 3rd quartile	4.1, 14.7		
Distance BG trap to nearest human house (m)	Range	0.1–17.6		0.44
	Mean	4.6		
	Median	3.1		
	1st quartile; 3rd quartile	0.3, 4.6		

*P*-values were calculated using the Kruskal–Wallis test or *t*-test with log-transformed outcome data

### Mosquito species from the study sites

**Fig. 2:**
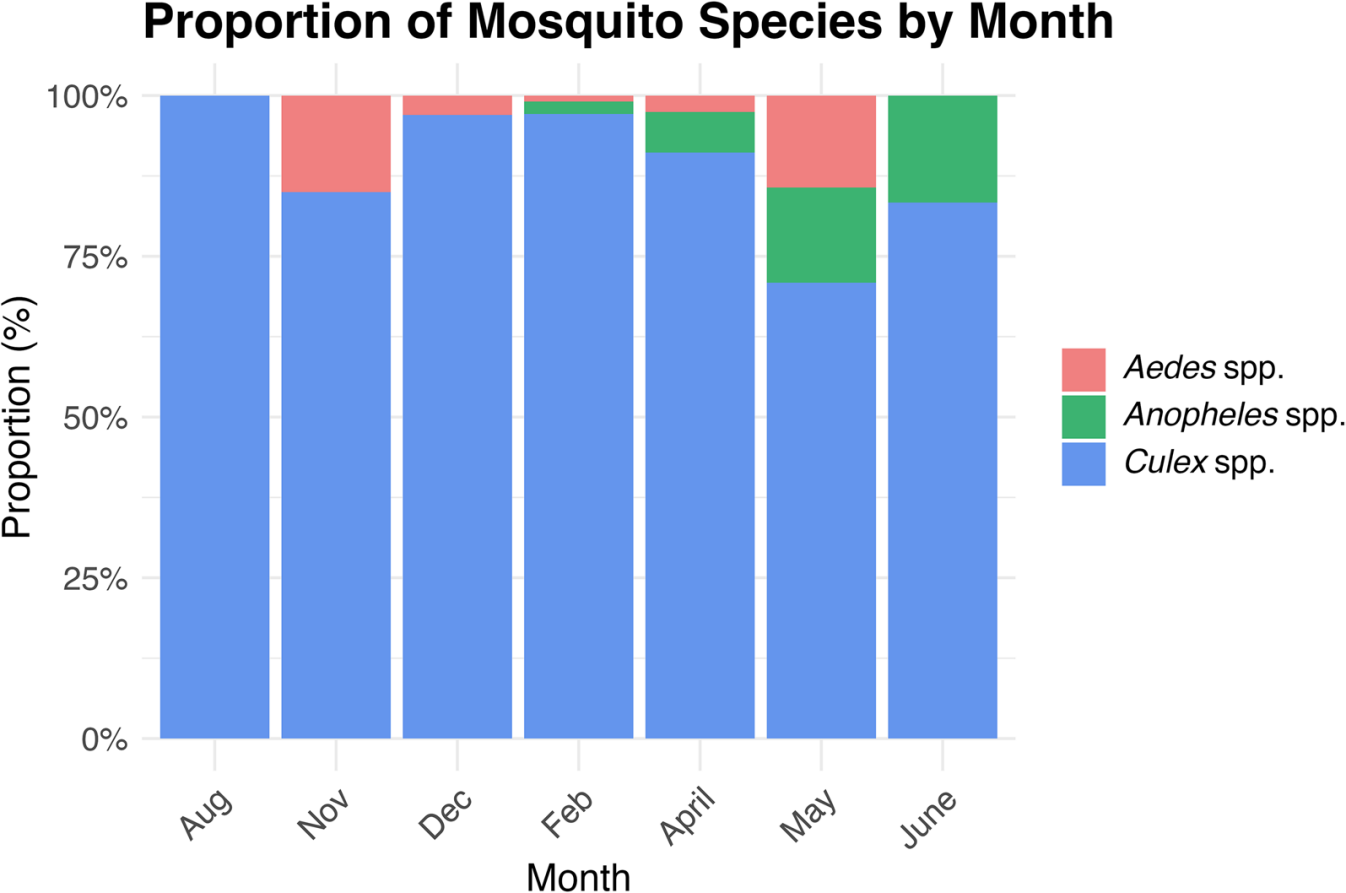
Proportion of mosquito species captured each month, normalized for the total number of mosquitoes per trapping month

Most mosquitoes caught in this study were *Culex* spp., and the mean proportion at each trapping event was 91.9% (range = 50–100%). The second most common were *Aedes* spp. (4.5%; range = 0–50%), followed by *Anopheles* spp. (3.6%; range = 0–50%). Change in species diversity by month is shown in Fig. 2. *Culex* spp. were present in all months, *Aedes* spp. from November to May (i.e. not the coolest months from June to August), and *Anopheles* spp. from February to June.

While the proportion of *Aedes* and *Anopheles* spp. captured in this study was low, the LULC of the household was a significant predictor for the presence of these species. Trapping events that occurred in shrubland had significantly more *Aedes* spp. (Kruskal–Wallis *χ*^2^ = 11.9, *df* = 2, *P* = 0.002), while trapping events that occurred in cropland areas had significantly more *Anopheles* spp. (Kruskal–Wallis *χ*^2^ = 6.9, *df* = 2, *P* = 0.03). All *Aedes* spp. in this study were morphologically identified as *Aedes aegypti*. Of the 25 *Anopheles* spp. mosquitoes trapped, 24 were identified as *Anopheles gambiae* and one as *Anopheles funestus*.

### Breeding sites

Standing water within the compound of the participating households was observed only at the start and end of the rainy season and was not significantly associated with mosquito abundance (Wilcoxon rank-sum test, W = 78, *P* = 0.42). Open water containers were observed in the compound at most trapping events (95%, 37/39), but larvae were found in only three containers across three events, in February (*n* = 2) and April (*n* = 1). The presence of active breeding containers with larvae was not significantly associated with mosquito abundance (Wilcoxon rank-sum test, W = 85, *P* = 0.08). Irrigation systems were observed adjacent to the household of some (31%, 12/39) trapping events, but this was also not significantly associated with mosquito abundance (Wilcoxon rank-sum test, W = 154, *P* = 0.82).

### Spatial and temporal associations with mosquito abundance

The unadjusted mosquito count data were over-dispersed (mean = 11.3, variance = 66.3), as were the outcome data adjusted per 24-h day (mean = 5.6, variance = 20.7).

LULC alone as a predictor resulted in poor model performance (AIC = 216.7, pseudo-*R*^2^ = 0.11, deviance = 42.7), while month produced a better model (AIC = 192.6, pseudo-*R*^2^ = 0.60, deviance = 37.2). However, including month and LULC in the same model resulted in significantly better fit (AIC = 177.1, pseudo-*R*^2^ = 0.77, deviance = 30.7), and accounting for LULC and month as an interaction term further increased the fit as measured by *R*^2^ and deviance, but decreased it as measured by AIC (AIC = 190.1, pseudo-*R*^2^ = 0.85, deviance = 19.7) The added complexity of the interaction model may not be justified according to the increased AIC. To increase the generalizability of model outputs, the categorical proxy of the month variable in models was exchanged with weather data.

### Comparison of weather station data

To select which weather station data to aggregate for trapping events, we compared the weather data between Ecowitt and Tinytag weather stations for each zone. Temperature and humidity data tracked well throughout most of the study period (Additional file 2, Fig. 1 a and b, top two panels). However, during periods of rainfall (Additional file 2, Fig. 1 a and b, bottom panels), the humidity fluctuations recorded by Tinytag are more extreme, and data recorded by the two stations further diverge. In a Pearson’s correlation analysis comparing mean parameters from the Ecowitt and Tinytag weather stations, temperature (*r* = 0.92,* P* < 0.0001) and RH (*r* = 0.69, *P* < *0.0001*) were highly correlated for the Kimana site, where the stations were mounted on the same pole. For the Rombo site, the stations were approximately 300 m apart and temperature between stations was highly correlated (*r* = 0.93, *P* < *0.0001*), while RH was only weakly correlated (*r* = −0.27, *P* < *0.0001*). For all modelling with weather data, only records from the respective Ecowitt weather stations were used.

### Association of mosquito abundance and weather variables

**Fig. 3:**
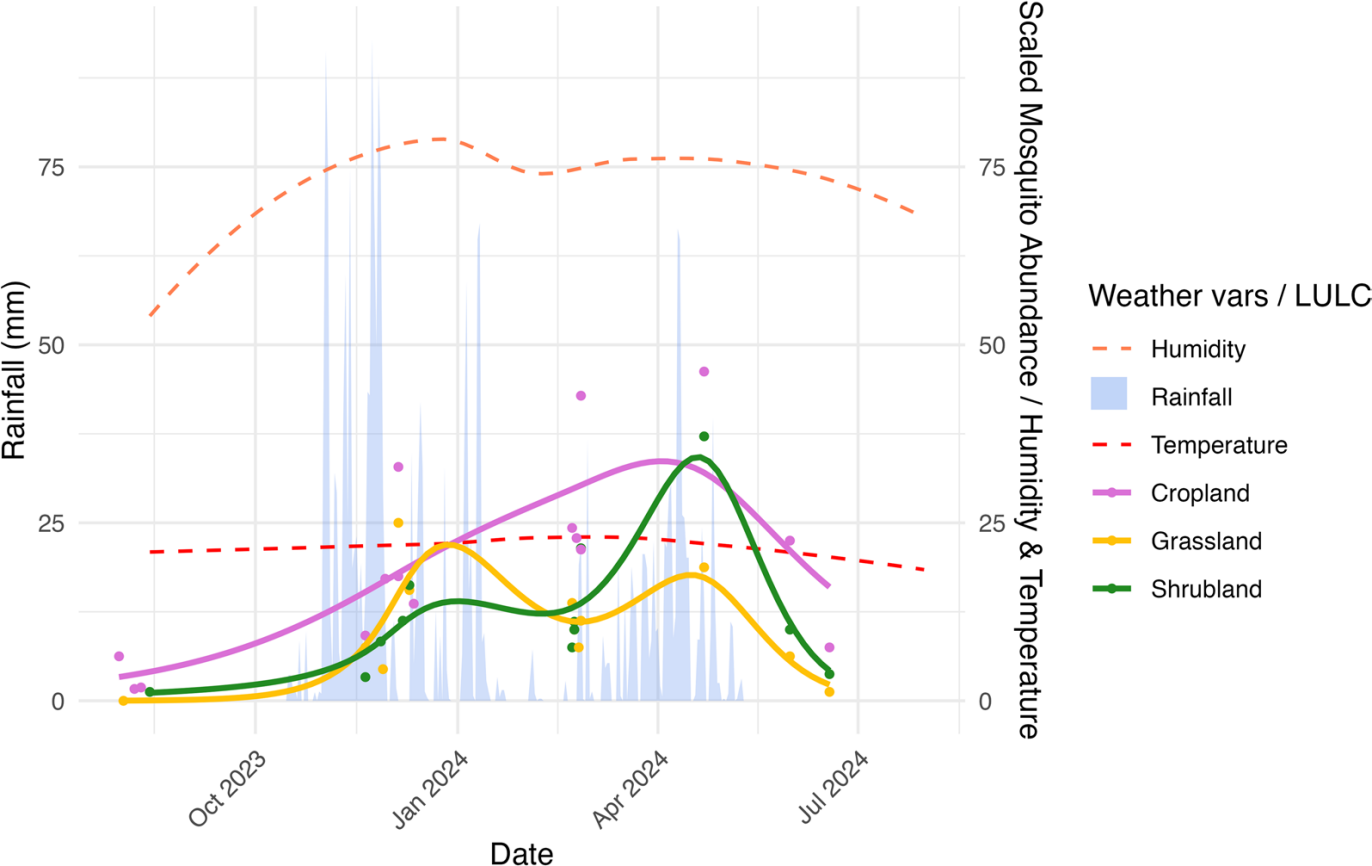
Summary of daily rainfall, temperature, and humidity over the study period with overlaid mosquito abundance trends grouped by LULC. Each point represents a trapping event at a household. A non-linear generalized additive model (GAM) line has been fitted for all data points in each LULC (cropland, grassland, shrubland). Units: rainfall (mm/day), temperature (°C daily mean), humidity (daily mean % RH). For visualization, trends in these variables have been smoothed. Vars: variables

Mosquitoes catch data over time are shown with overlaid Ecowitt weather station data in Fig. 3, where weather variables are averaged across the Kimana and Rombo stations. Temperature, humidity, and rainfall varied significantly across trapping months, as did temperatures recorded at each station (Table 2). While mosquito abundance in grassland and shrubland followed a bimodal pattern aligned with rainfall, abundance in cropland continued to increase between rainy seasons (Fig. 3). The month with the highest rainfall was November 2023, but the peak mosquito count in cropland occurred in February 2024, after two consecutive rainy seasons.

**Table 2:** Comparison of weather parameters for the matched Ecowitt stations (Rombo and Kimana) across all trapping events, aggregated by zone, LULC, and trapping month

		Temperature (°C) averages in the prior		Rainfall (mm) totals in the prior		Humidity (% RH) averages in the prior	
		2 weeks	1 month	2 weeks	1 month	2 weeks	1 month
Vector study variables		Avg (range)	Avg (range)	Avg (range)	Avg (range)	Avg (range)	Avg (range)
Study zone	Kimana	21.6 (18.9–23.4) *P* = 0.08	21.4 (20.9–22.8) *P* = 0.23	115.0 (0–285) *P* = 0.20	230 (0–557) *P* = 0.58	72.8 (62.2–84.1) *P* = 0.87	73.0 (65.7–82.0) *P* = 0.31
	Rombo	22.5 (20.1–24.3)	22.2 (19.9–23.8)	121 (0–255)	255 (0–508)	75.7 (67.1–83.1)	78.2 (70.6–85.3)
	Loitokitok	21.2 (19.0–24.3)	21.3 (18.8–23.8)	49.1 (0–155)	128 (0–354)	72.9 (61.0–84.2)	73.8 (65.3–81.4)
Ecowitt station for trapping event	Kimana	21.1 (18.9–23.4) *P* = 0.002	21.1 (18.8–22.8) *P* = 0.007	67.8 (0–285) *P* = 0.19	160 (0–557) *P* = 0.71	73.2 (61.0–84.2) *P* = 0.82	73.9 (65.3–82.0) *P* = 0.45
	Rombo	22.9 (20.1–24.3)	22.5 (19.9–23.8)	99.2 (0–255)	207 (0–508)	74.1 (67.1–83.1)	76.6 (70.1–85.3)
LULC	Grassland	21.7 (19.0–24.3) *P* = 0.85	21.6 (19.0–23.8) *P* = 0.77	81.2 (0–282) *P* = 0.997	175 (0–544) *P* = 0.94	73.5 (61.0–84.2) *P* = 1.00	74.6 (65.3–85.3) *P* = 1.00
	Shrubland	21.7 (19.0–24.3)	21.6 (18.9–23.8)	72.0 (0–240)	168 (0–500)	73.5 (61.7–84.2)	74.5 (65.6–84.9)
	Cropland	21.4 (18.9–24.3)	21.3 (18.8–23.8)	74.6 (0–285)	174 (0–557)	73.4 (61.7–84.2)	74.6 (65.6–85.3)
Month of trapping event	August (2024)	19.2 (18.9–20.1) *P* < 0.0001	19.1 (18.8–19.9) *P* < 0.0001	0 (0–0) *P* < 0.0001	0 (0–0) *P* < 0.0001	70.8 (69.4–72.1) *P* < 0.0001	71.2 (70.6–72.6) *P* < 0.0001
	November 2023	20.7 (20.6–20.7)	21.1 (20.7–21.4)	197 (124–285)	409 (289–557)	82.9 (82.2–84.1)	77.9 (74.6–82.0)
	December 2023	21.6 (21.3–21.9)	21.3 (20.9–21.6)	182 (33.5–255)	439 (342–508)	80.4 (76.6–83.1)	83.4 (80.5–85.3)
	February 2024	23.7 (23.3–24.3)	23.2 (22.7–23.8)	3.54 (0–17.5)	10.1 (6.4–21.1)	64.2 (61.0–67.7)	67.7 (65.3–70.8)
	April 2024	21.1 (21.1–21.1)	21.6 (21.6–21.6)	155 (155–155)	339	84.2	81.4
	May 2024	20.6 (20.6–20.6)	20.9 (20.9–20.9)	0 (0–0)	18.2	74.4	77.1
	June 2024	20.0 (20.0–20.0)	20.3 (20.3–20.3)	0 (0–0)	0	68.3	71.0

*P*-values were calculated using the Kruskal–Wallis test. *Avg* average, *Hum* humidity, *Temp* temperature, *LULC* land use and land cover, *RH* relative humidity

**Fig. 4:**
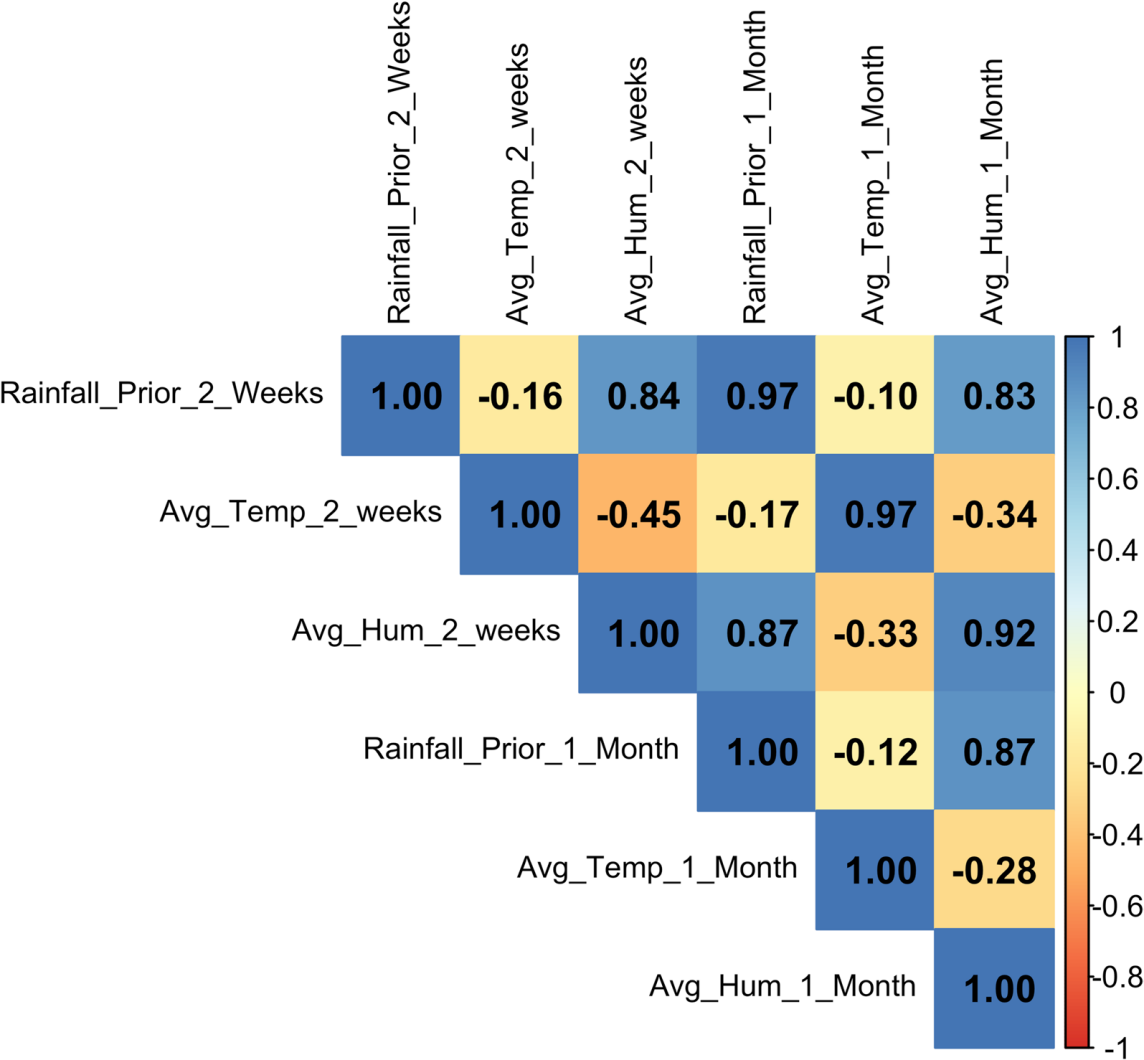
Pearson’s correlation coefficient matrix of weather station variables. Hum, humidity; Temp, temperature; Avg, average. Values between 0.7 and 1 indicate high correlation, 0.4–0.7 moderate, 0.2–0.4 weak, and < 0.2 very weak or not correlated

A Pearson’s correlation matrix was used to assess weather variable collinearity (Fig. 4). As expected, 2-week and 1-month values were highly correlated. Temperature and humidity (2-week) were moderately negatively correlated (*r* = −0.45, *P* = 0.004), while rainfall and humidity were strongly correlated (*r* = 0.84, *P* < 0.001) and not included in the same model.

A summary of all metrics from multivariable models is presented in Additional file 1. In general, inclusion of the LULC variable always improved model fit, driven by cropland, demonstrating a significantly higher mosquito abundance. Weather data from the prior 1 month resulted in a better fit than weather data from the prior 2 weeks. The model including LULC, temperature in the prior 1 month, and humidity in the prior 1 month best predicted mosquito abundance (AIC = 195.3, pseudo-*R*^2^ = 0.53, deviance = 36.4). A similar model that substituted rainfall for humidity performed comparably (AIC = 198.3, pseudo-*R*^2^ = 0.49, deviance = 37.9), suggesting that either metric may serve as a useful environmental predictor, practically speaking. Both models are summarized in Table 3.

**Table 3:** Summary of the top two best-fit models with weather data to predict mosquito abundance. Weather parameters were computed as averages in the 1 month prior to sampling

	Total mosquito abundance ~ LULC + temperature + humidity (*Best-fit model with weather data*)			Total mosquito abundance ~ LULC + temperature + rainfall		
Predictors	Incidence rate ratios	Confidence interval	*P*-value	Incidence rate ratios	Confidence interval	*P*-value
(Intercept)	0.00	0.00–0.00	< 0.001	0.00	0.00–0.00	< 0.001
LULC [Shrubland]	1.10	0.68–1.80	0.696	1.10	0.68–1.80	0.696
LULC [Cropland]	2.07	1.33–3.25	0.001	2.07	1.33–3.25	0.001
Avg Temp 1 month	1.54	1.30–1.84	< 0.001	1.54	1.30–1.84	< 0.001
Avg Hum 1 month	1.06	1.03–1.09	< 0.001	1.06	1.03–1.09	< 0.001
Rainfall prior 1 month				0.00	0.00–0.00	< 0.001
Observations	39			39		
AIC	195.2			198.3		
Deviance	36.4			37.9		
McFadden’s pseudo-*R*^2^	0.53			0.49		

*Avg* average, *Hum* humidity, *Temp* temperature, *LULC* land use and land cover. Grassland is the reference category for the LULC variable

A scatter plot of the observed versus predicted counts for the best-performing model was evenly distributed around the 1:1 line. While models using weather variables provided acceptable predictive performance, models using month consistently outperformed them. This is highly site-specific, however, limiting its generalizability, and weather variables are preferred.

## Discussion

This study demonstrated the importance of spatial and temporal components for predicting mosquito abundance in semi-pastoral lowland areas. Overall, mosquito abundance was lower than other areas in Kenya with greater vegetation, rainfall, and population density [38, 41]. Yet, even during very dry periods, we caught few *Culex* spp. at the household level, suggesting that if RVFV is maintained endemically via livestock-livestock transmission, this would likely be a *Culex*-driven process. Cropland areas may also be important for maintaining local populations of mosquitoes in between rainy seasons. Low mosquito abundance overall suggests that other factors, such as extensive livestock production and movement, are important to support ongoing low-level transmission [42, 43].

Spatially, inclusion of LULC in multivariable models consistently improved model fit. Notably, grassland and shrubland households experienced a bimodal distribution of mosquito abundance between the short (Oct–Nov) and long (March–May) rainy seasons (Fig. 3). The lack of a decrease at cropland sites during this dry time likely reflects widespread use of irrigation, which provides stable breeding habitats for mosquitoes. We found significantly more *Anopheles* spp., most of which were *An. gambiae*, at cropland households (Kruskal–Wallis *χ*^2^ = 6.9, *P* = *0.03*), which is potentially linked to irrigation canals with clean shallow water for mosquito breeding. This pattern aligns with findings from western Kenya, where *An. gambiae* utilized both temporary and permanent water bodies, with breeding site availability shifting in response to rainfall intensity [44]. Similarly, a study in northern Kenya found higher mosquito densities in irrigated areas across both wet and dry seasons, along with increased RVFV seroprevalence compared to non-irrigated pastoral zones [32]. The presence of *Anopheles* spp. is also highly relevant for future malaria risk, and while the Loitokitok sub-county is currently low-risk, expansion of irrigated agricultural has shown to decouple seasonal weather patterns and VBD risk [45] [46]. These trends must be assessed with regard to consistent trapping methods, as another nearby study in Kajiado County reported higher abundance and additional species that we did not identify in our study [47].

While RVFV can utilize many mosquito species for transmission, *Aedes* spp. tend to be the most competent in laboratory studies [48, 49]. We observed the highest proportion at the start of the short rains (November) and end of the long rains (May), with significantly more *Ae. aegypti* in shrubland, which aligns with their resting preference. No floodwater *Aedes* spp. were caught even though parts of the study site were flooded following 2023–2204 El Niño rains. The ecological interface between shrubland and cropland, present across our study area, may support increased vector diversity, and is more broadly indicative of emerging disease risks on the edges of agricultural habitats where environments that are highly human-managed stand beside areas which are relatively undisturbed [50]. In addition, human behaviours may also impact the presence of these vectors close to homesteads. We screened all open water containers at each trapping event and found larvae only towards the end of the rainy seasons, likely reflecting water storage behaviour in anticipation of scarcity in upcoming dry periods. Overall, we found very little evidence of household-level mosquito breeding, and the presence of larvae was not associated with total abundance (Wilcoxon rank-sum test, W = 85, *P* = 0.08), which points towards either unnoticed or inaccessible breeding sites or breeding outside of the household. Larval source management (LSM) at the end of the rainy season, when rising temperatures accelerate development, should target both households and adjacent irrigation areas as part of IVM strategies. Ultimately, different species may be important in driving endemic RVFV transmission at different times of the year and in different ecological areas.

Temporally, mosquito abundance rose steadily with increasing rainfall and humidity but declined sharply once rainfall ceased. In our study, rainfall subsided completely in mid-May 2024 (Fig. 3), coinciding with the highest shared proportion of *Aedes* and *Anopheles* spp. (Fig. 2) and by June, no *Aedes* spp. were captured, suggesting that they may die off first in dry conditions. While we did not perform a temporal species-specific analysis due to low numbers, other studies have shown that links between mosquito abundance and the environment are species-specific [51]. A study in Senegal on malaria vectors showed that mosquitoes are opportunistic in complex sylvo-pastoral areas, and their biting preferences depend on the availability of hosts and presence of standing water [51, 52]. This complexity, even within a single genus, further supports the decision to analyse total mosquito abundance regarding RVFV transmission risk.

Our multivariable model showed that weather variables, particularly temperature and humidity in the prior 1 month, were strong predictors for mosquito abundance. This is particularly relevant when the period between rainy seasons is short, such as during the 2023–2024 El Niño event, which prolonged the short rains [53]. Temperature was consistently retained in the best-fit models, and humidity slightly outperformed rainfall as an additional covariate, suggesting that both are reliable predictors. The Tinytag data loggers showed inconsistent humidity readings during heavy rainfall, so Ecowitt data were used in all models. This station proved highly cost-effective compared to other solar-based systems that transmit data remotely, and placing these stations at health facilities ensured proper infrastructure and monitoring while reinforcing the link between environmental and human health.

Future efforts focused on RVFV vectors could address our study limitations. First, integrating fieldwork in another study led to inconsistent trapping intervals. Although we adjusted for the duration in analyses, differences between morning and afternoon catches may have impacted our results. Increasing the number of households sampled per LULC type and directly comparing cropland-adjacent livestock-owning households would improve statistical power and clarify spatial patterns. We assumed that transmission of RVFV was primarily at the household, and we did not trap mosquitoes outside of compounds. Yet, studies have shown that *Aedes* spp. often proliferate in open areas [54], and this could be explored in future studies, especially where households may be denser and span multiple LULC types. We also relied on BG traps and visual larval inspection, which may have biased the genera observed as well as the total abundance. Our goal was to compare abundance trends across time and sites, but future studies should include additional trap types to enable direct comparisons, as the limited trapping approach may have impacted the power of predictive models. We did not complete molecular identification of mosquitoes, which would have added to the information gained in this study. Finally, spatial variation in lag times between environmental conditions and mosquito abundance should be further explored to improve predictive models.

Overall, this study links RVFV-competent mosquito abundance broadly to weather patterns and LULC in semi-pastoral settings, as these factors are likely important for sustaining endemic RVFV transmission. While our study is limited in geographical and temporal scale, this approach could be expanded to include more traps and additional sites, helping to clarify how weather and land use shape mosquito abundance. Our results can contribute to endemic VBD risk assessments and support targeted IVM strategies, particularly those involving community participation and engagement with the agricultural sector.

## Conclusions

This study demonstrates that with relatively simple weather data and repeated mosquito trapping over time across different LULC areas, mosquito abundance trends can be predicted at the household level in semi-pastoral settings in Kenya. Cropland areas had the highest mosquito abundance and may sustain mosquito populations between rainy seasons, with temperature and humidity from the prior 1 month better predicting abundance than shorter time frames. The continued conversion of natural grassland and shrubland to irrigated cropland is likely to increase total mosquito abundance in semi-pastoral areas and impact VBD dynamics. This study provides the basis for a potential mechanism to monitor trends over time and target IVM strategies aimed at reducing vector abundance and controlling complex VBDs such as RVF in semi-pastoral areas.

## Supplementary Information

Additional file 1. Supplementary Excel file containing comparison of the multivariable model metrics.

Additional file 2. Supplementary Figure S1 (a, b). Comparison of weather data recorded by Ecowitt and Tinytag stations.

## Data Availability

Data supporting the main conclusions of this study are included in the manuscript.

## Abbreviations

AIC: Akaike information criterion
BG: Biogents
CHIRPS: Climate Hazards Group InfraRed Precipitation with Station data
GAM: Generalized additive model
ILRI: International Livestock Research Institute
IVM: Integrated vector management
KEMRI: Kenya Medical Research Institute
LULC: Land use and land cover
*r*: Pearson correlation coefficient
RH: Relative humidity
*R*^2^: Coefficient of determination
RT-PCR: Reverse transcription polymerase chain reaction
RVF: Rift Valley fever
RVFV: Rift Valley fever virus
UV: Ultraviolet
VBD: Vector-borne disease
