# Supplementary material for: Seasonal variation in mosquito abundance and environmental predictors in semi-pastoral southern Kenya: implications for endemic Rift Valley fever
Source: Parasit Vectors. 2025 Nov 27;19:1. doi: 10.1186/s13071-025-07122-1 (PMC12764129; doi:10.1186/s13071-025-07122-1)
Supplement: Supplementary file 2 — Additional file 2. Supplementary Figure S1 (a, b). Comparison of weather data recorded by Ecowitt and Tinytag stations. [file 13071_2025_7122_MOESM2_ESM.pdf]

Additional file 2– Comparison of weather station data

Figure 1: Comparison of parameters from Kimana (a) and Rombo (b) Ecowitt and Tiny tag weather stations

a)

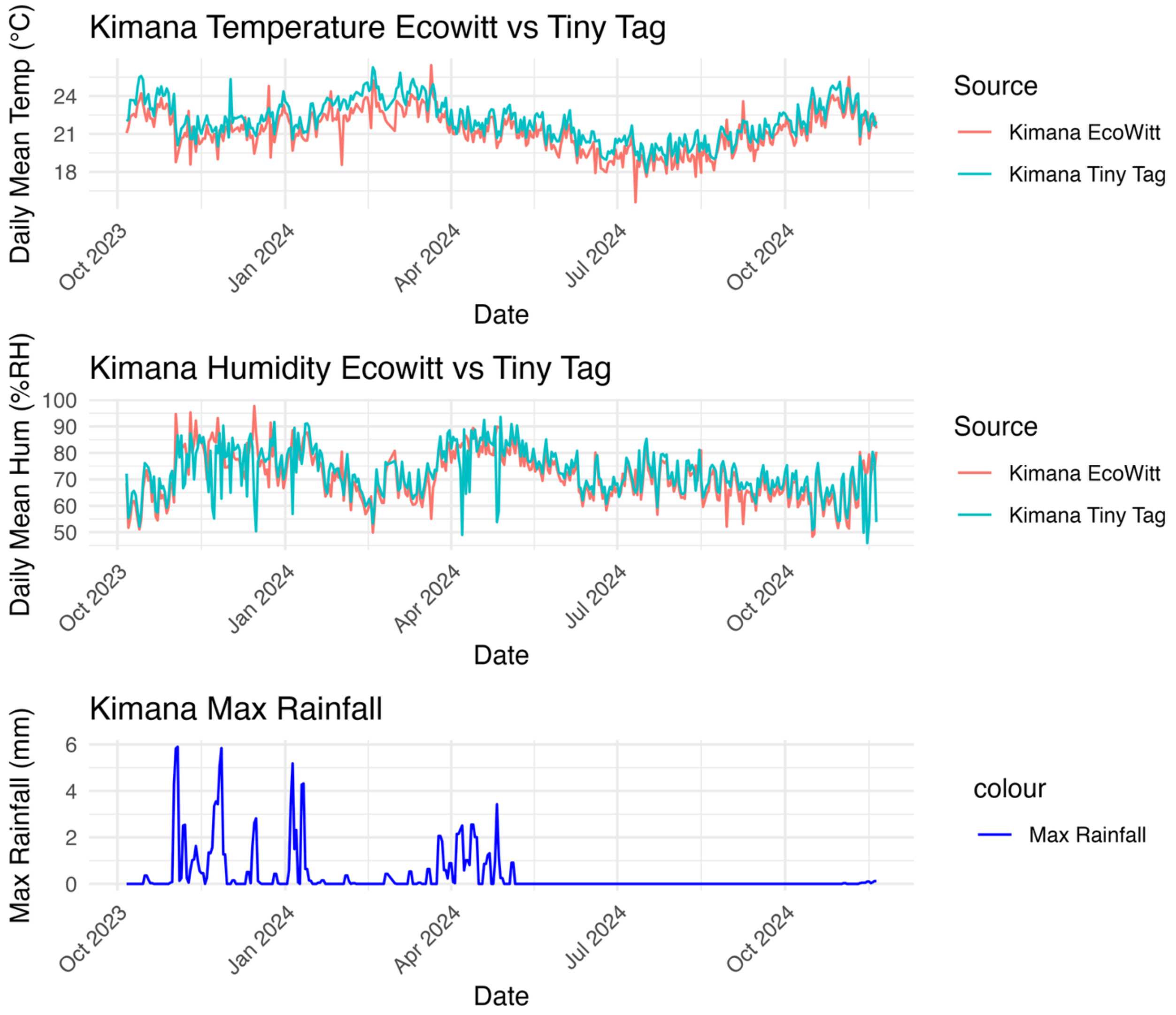

b)

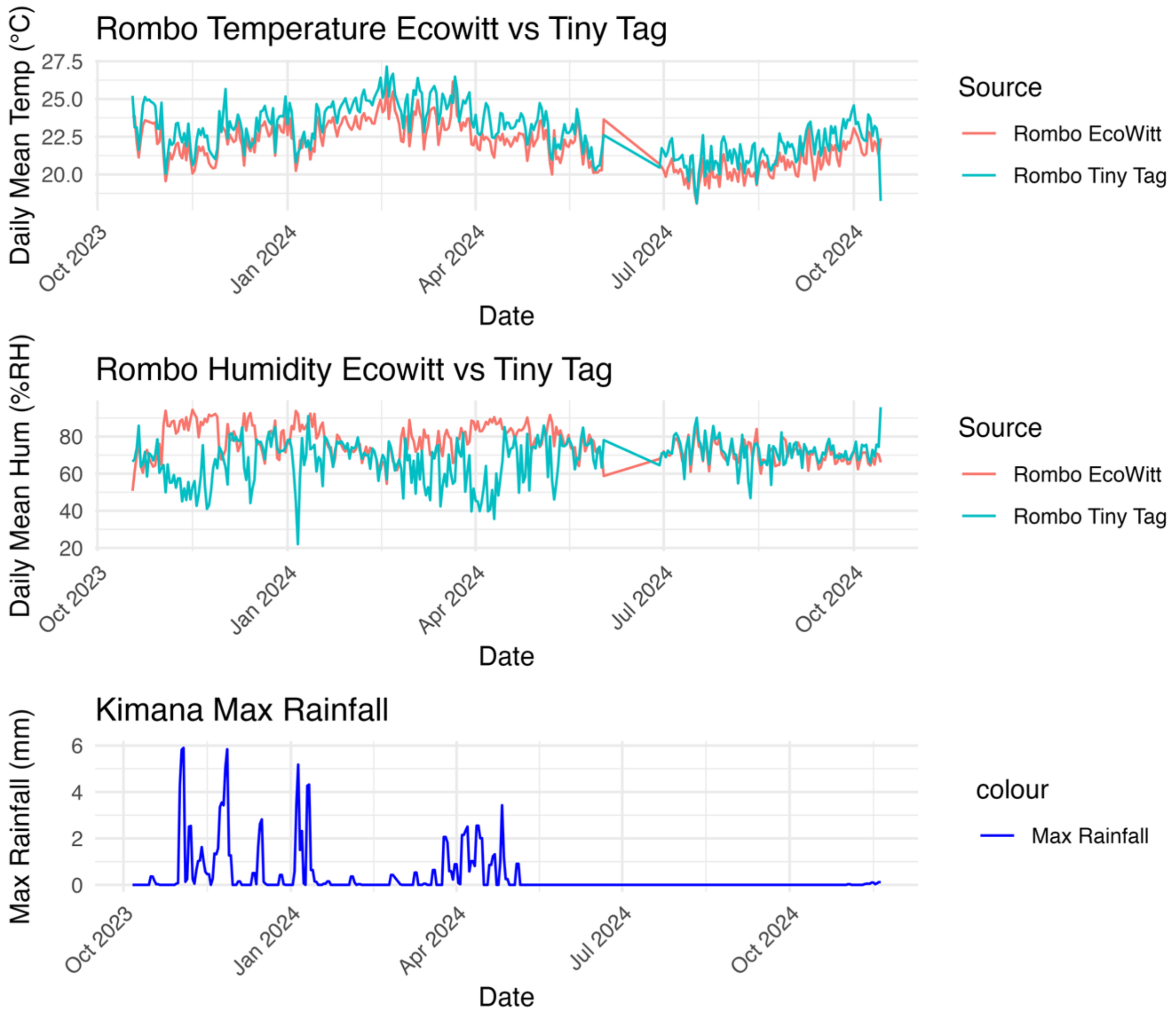

Note: Missing June 2024 data from Rombo Ecowitt, so therefore, the Tiny Tag data has also been removed for consistent comparison. Temp: temperature, Hum: humidity
